# 

**Published:** 1910-04-23

**Authors:** 


### THE GRADUATES' MEDICAL SCHOOLS.

DIARY FOR THE WEEK, APRIL 25 to APRIL 30.

### LONDON SCHOOL OF CLINICAL MEDICINE, Seamen's Hospital, Greenwich, S.E.

At 2.15 p.m.—**April 25**, Mr. William Turner, **Some Surgical Principles and their application.**
4 p.m.—**April 28**, **Epileptic Manifestations.**

### MEDICAL SOCIETY OF LONDON, Chandos Street, Cavendish Square, W.

At 8.30 p.m.—**April 25**, Mr. Frederick Eve, **Some Points Regarding the Symptoms and Diagnosis of Gastroptosis, with the end results of Operative Treatment.** Dr. R. Hutchison, **The Treatment of Gastroptosis.**

### CENTRAL LONDON THROAT AND EAR HOSPITAL, Gray's Inn Road, W.C.

At 3.45 p.m.—**April 26**, Dr. Andrew Wylie, **Larynx.**
3.45 p.m.—**April 29**, Dr. Andrew Wylie, **Larynx.**

### ROYAL SOCIETY OF MEDICINE, 15 Cavendish Square, W. (Temporary address during building.)

At 8 p.m.—**April 25**, **Odontological Section:**
At 15 Cavendish Square, W.
*Paper*: Mr. A. Hopewell Smith: "Two odontoceles and some other cysts."
*Casual communications.*—Mr. C. Schelling: (a) "Two cases of suppuration in the maxilla, and (b) a case of neuralgia due to impacted lower wisdom teeth, in all of which skiagrams helped the diagnosis." Mr. Herbert Tilley: "Two cases of suppurating dental cysts invading the antrum." Mr. W. Rushton: "A case of painful attrition."

5.30 p.m.—**April 26**, **Medical Section:**
At 15 Cavendish Square, W.
*Paper*: Dr. J. E. Squire, C.B.: "Hospital infection of tuberculosis as exemplified by the records of the Resident Staff of the Mount Vernon Hospital for the past fifteen years."

4.30 p.m.—**April 28**, **Neurological Section:**
At the National Hospital, Queen Square, W.C.
Clinical meeting.

ANNOUNCEMENT.—Surgical Section: The next two meetings will be held at the Botanical Theatre at University College, on Tuesday, May 10, and Tuesday, June 14, at 5.30 p.m., when a discussion on "The present position and treatment of syphilis" will be opened by Mr. Ernest Lane. Sir Jonathan Hutchinson, Prof. Alexander Flemming, and Mr. Arthur Shillitoe are amongst those who have promised to take part. Gentlemen wishing to speak in the discussion are requested to send their names as soon as possible to the Hon. Secretary, Mr. J. Hutchinson, 1 Park Crescent, W.

### THE POST-GRADUATE COLLEGE, West London Hospital, Hammersmith, W.

At 10 a.m.—**April 25**, Demonstration by Surgical Registrar.
10 a.m.—**April 28**, Demonstration by Surgical Registrar.

### MEDICAL GRADUATES' COLLEGE AND POLYCLINIC, 22 Chenies Street, W.C.

At 4 p.m.—**April 25**, Dr. J. M. H. MacLeod, **Clinique (skin).**
5.15 p.m.—**April 25**, Dr. David Ferrier, **The Cerebral Circulation.**
4 p.m.—**April 26**, Dr. R. Hutchison, **Clinique (medical).**
5.15 p.m.—**April 26**, Dr. F. J. Smith, **Ulcer of Stomach and Duodenum.**
4 p.m.—**April 27**, Mr. Chas. Ryall, **Clinique (surgical).**
5.15 p.m.—**April 27**, Mr. Herbert Fisher, **Concomitant Strabismus.**
4 p.m.—**April 28**, Dr. Porter Parkinson, **Clinique (medical).**
5.15 p.m.—**April 28**, Dr. Alex. Morison, **Aneurysm of the Thoracic Aorta.**
4 p.m.—**April 29**, Mr. R. E. Bickerton, **Clinique (eye).**

The provincial sessional meeting of the Royal Sanitary Institute, on Friday, April 29, will be held in the Guildhall, Worcester, at 7.30 p.m., when a discussion will take place on "Teaching of Sanitary and Domestic Science in Schools," to be opened by G. H. Woollatt, Ph.D., F.I.C. The programme for the sessional meetings are as follows: Friday, April 29, 1910, 7.30 p.m., meeting in the Guildhall, Worcester. Saturday, April 30, 9.30 a.m., meet at the Guildhall, and proceed to Messrs. Edward Webb & Sons' horse-hair factory, and Messrs. Dent, Allcroft and Co.'s glove manufactory; 10.30 a.m., proceed to the Royal Porcelain Works; 11.30 a.m., visit Messrs. Thomas and Son, Pump Works, Wyldes Lane, returning *via* Friar Street to inspect ancient buildings of historical and archæological interest, when it is hoped some local gentlemen will act as guides; 1 p.m., luncheon at Star Hotel; 2.15 p.m., visit to the Cathedral and Deanery; 4 p.m., proceed by way of West end of Cathedral and ruins of ancient hospital to Cathedral promenade, over Worcester Severn Bridge, to the Corporation Electricity Works, Hylton Road, where members will be shown round the station by C. M. Shaw, Esq., electrical engineer.

Mr. F. M. O'Reilly, headmaster of the St. James's Boys' School at Athboy, has made some interesting experiments in school disinfection, using Sanitas as a medium, and reports the results in the *Irish School Weekly*. The period over which the disinfection was carried out could not have been more favourable from an experimental point of view—diphtheria and whooping cough were prevalent in the district, and some of the schools were closed in consequence. During the entire twelve months there has not been a single case of illness among the pupils. The total cost amounted to only a few shillings. The only appliance used was a watering can, filled with dilute "Sanitas Okol." The floors were sprinkled occasionally during the week. On Friday evenings they were thoroughly soaked, and the windows were then closed, to allow the vapour to impregnate the walls, &c. Mr. O'Reilly is making a report of this experiment to the International Society for the Promotion of School Hygiene.

The Lord Mayor, President, took the chair at the annual public meeting of the Hospital Saturday Fund last Saturday at the Mansion House. The Hon. H. L. W. Lawson, M.P., Treasurer, moved the adoption of the report in the absence, through illness, of Sir Savile Crossley, Chairman of the Fund. He pointed out that the Fund had relieved the working classes of London from what was something of a reproach—that they did not appreciate the duty of self-insurance against the risks of accident and sickness to the same extent as was done by their fellows in the industrial centres of the north and elsewhere. Comparing the figures for 1909 with those for 1897—the first year in which the street collection was discontinued—he said that the income had risen from £20,138 to £30,662, the benefits issued from 26,580 to 53,179, while the expenses had fallen from 12.5 per cent. to 7.9 per cent. One-sixth of the total income of the Fund was spent in the crusade against consumption. In the dentistry department the number of cases helped had gone up from 174 in 1897 to 1,892 in 1909. Mr. Montagu F. Hopson, dental surgeon to Guy's Hospital, moved a resolution approving the action of the Fund in providing assistance to those needing dental treatment. He emphasised the importance of providing dental treatment for children, and said that although we could not hope to eradicate dental disease in the course of a generation or two, yet the serious general consequences which ensued could be prevented altogether, provided that proper treatment were undertaken sufficiently early. The resolution was carried.

### Gifts and Bequests.

A sum of £200 has been received from Mr. J. Wallis Goddard for the Extension Fund of Leicester Infirmary.

Miss Charlotte Lea, of Leamington, Warwick, has left £100 to the Birmingham Medical Mission.

Miss Mary Matthews, of Sheffield, has left £500 to the Children's Hospital, Sheffield.

The Hon. Mrs. Grosvenor Hood, of Freshwater, Isle of Wight, has left £100 to the Royal Ryde Infirmary.

Mr. John Clarke has left £100 each to the Sheffield Royal Hospital and the Sheffield Royal Infirmary, and £50 to the Free Hospital for Children, Western Bank, Sheffield.

The Clothworkers' Company has given £100 to Queen Charlotte's Hospital and £50 to the East London Nursing Society.

The Treasurers of the Middlesex Hospital have received a further donation of £105 from Messrs. Crosse and Blackwell.

Under the will of Mrs. Mary Anne Jones, of Leicester, a legacy of £100 free of duty is bequeathed to the Leicester Infirmary.

The Leicester Infirmary has received from the Committee of the Local Co-operative Society £100 for the extension fund in commemoration of the Society's jubilee year, 1910.

The late Colonel Trevenen James Holland, C.B., of Tunbridge Wells, a Mutiny veteran, has bequeathed £500 to the maintenance fund of the Eye and Ear Hospital, Tunbridge Wells.

Mr. John Foster, of Coombe Park, Whitchurch, Oxon., deputy-chairman of the Great Eastern Railway Company, left £500 each to the Whitby Cottage Hospital and the Whitby Seaside Home, and £250 each to the Surgical Aid Society and the Surgical Appliance Society.

The Rev. Edward Henry Rogers, of Pewsey, Wilts, has left two cottages and land at Oare, valued at £400, upon trust for sale, and to hold the proceeds upon trust to provide a trained nurse for the parishes of Oare and Huish; £300 to the Royal Eye Hospital, Southwark; and £200 to the Brompton Cancer Hospital.

Among the latest contributions received at the Bank of England for King Edward's Hospital Fund for London are the following annual subscriptions: Messrs. Speyer Bros., £250; Duke of Norfolk, £50; Lord Wemyss, £50; "A. G. S.," £25; Mr. John Aird, £25; Mrs. John Aird, £25; Captain J. C. Miller, £25; Lord Lister, £20. Donation: Messrs. Crosse and Blackwell, Ltd., £105.

Mrs. Kate Minnie Russel, of Halstead, Kent, has left £200 each to the Royal Hospital for Incurables, Putney, and the Hip and Spine Diseases Hospital for Children. She has also left to the Victoria Hospital for Children, Chelsea, her freehold residence Koonowla, Biggin Hill, Cudham, Kent, with furniture and effects belonging thereto, desiring that it may be used as a convalescent home, and £5,000 upon trust to apply the same for the maintenance of the said residence as a convalescent home.

The Treasurer of St. Bartholomew's Hospital acknowledges the following further contributions towards the Special Appeal Fund: Anonymous, £200; Mr. Walter Leaf, £105; Mr. John T. Christopher (second donation), £52 10s.; Messrs. Emile Erlanger and Co., £52 10s.; Mr. R. B. Longridge (second donation), £50; "R. X.," £26; the Great International Plate Glass Insurance Co., Ltd., £25; Messrs. Henry Kendall & Sons, £25; Sir R. W. Buchanan Jardine, £25; Messrs. E. A. de Pass and Co., £10; Sir W. J. Farrer, £10.

### Jottings.

The Lord Mayor, supported by the Sheriffs, presided over the Finsbury Dispensary festival banquet, held at De Keyser's Hotel, last week.

On April 12 the Edward Terry Dramatic Club played "Niobe" at the Cancer Hospital, Brompton. The piece was capitally acted and much enjoyed.

Thanks to an anonymous gift of £2,000, which has enabled the trade debts to be paid, the Kensington and Fulham General Hospital, which has been closed for some time for want of money, has now been reopened.

The new wards at Steevens' Hospital, Dublin, have now been opened. Their furniture was largely paid for by the proceeds of a matinée given on St. Patrick's Day. The new chapel contains a marble slab to commemorate Madame Steevens, the foundress.

Earl and Countess Fortescue, Lady de Ros, Lady Agneta Montagu, Lady Wallscourt, Lady Mary Dawson, and Lady Willshire were present last week at the New Royalty Theatre for the dramatic performance in aid of the East London Hospital for Children at Shadwell.

The monthly meeting of the Executive Committee of the Worcester General Infirmary, held on April 18, being the first since the annual meeting, was mainly occupied with the election of officers. The chairman and vice-chairman were re-elected. An increase in salary was granted to the lady dispenser. Dr. Crowe, on behalf of the hon. medical staff, reported that no suggestion could be made to reduce the expenditure on drugs, dressings, and surgical instruments. Any alteration likely to occur would be on the side of increase. It was announced that after payment of the monthly accounts, the balance against the infirmary would be £1,158 11s. 9d., unless in the meantime more support were received.

The Weir Hospital Charity Committee of the Wandsworth Borough Council has taken counsel's opinion on the judgment of Mr. Justice Eve with reference to the Council's petition in opposition to the scheme of the Charity Commissioners for the administration of the Weir Hospital Charity, and will recommend the Council to proceed with the appeal against the judgment. The dispute is over the administration of a sum of about £50,000 bequeathed by the late Mr. Benjamin Weir, of Streatham, formerly Chairman of the Wandsworth Board of Works. The Borough Council contend that the money was devised to provide a hospital for the people of Streatham, whereas the Commissioners propose to hand it over to the Bolingbroke Hospital, in the borough of Battersea.

A tablet over the bed which was endowed by the Emperor of Japan at the Seamen's Hospital, Greenwich, has been unveiled by the Japanese Ambassador. Mr. Percival A. Nairne, chairman of the committee of management, presided over a meeting, which was addressed by the Japanese Ambassador, who expressed, on behalf of his Emperor, his gratitude that such an institution as the Seamen's Hospital existed. It was the first opportunity that had come in his way of publicly recording the indebtedness of the Japanese nation to the Hospital for the care and attention that was bestowed on Japanese seamen admitted to its wards. Mr. William Turner, senior surgeon, expressed the appreciation of the honorary medical staff for the bed and the tablet given by the Emperor of Japan. Admiral Sir John Durnford then accepted the gift of the tablet on behalf of the Society, and expressed its appreciation of the Emperor's good will. After the speeches a reception was held.

### THE QUESTION BOX.

**SPECIAL NOTICE.—Questions of interest affecting the honorary medical staff and hospital administration in all departments are invited. When any point arises we hope our readers will formulate it in a question and so co-operate to make this column of real value and interest. In this way the experience of the best-managed hospitals should be made helpful to the whole hospital world. We shall welcome the contribution of both questions and answers.**

**N.B.—The publication of an answer in this column does not necessarily preclude the publication of subsequent answers to the same question, for a question may involve points which require to be threshed out and fully discussed. Answers, if published, will be paid for at scale rates.**

### OUT-PATIENT CASUALTY ADMITTANCES.

Question: Should accidents admitted as in-patients from the casualty department be counted as casualties?

### A Birmingham View.

By Mr. Howard J. Collins, Birmingham General Hospital.

In my opinion no case should be reckoned twice either when examined in the casualty department and admitted as an in-patient, or when examined in the casualty department and transferred to the out-patient department, or when examined in one department of the out-patients and transferred to another special department.

The object of the publication of the number of cases treated obviously is to show the number of individuals who receive the assistance of the hospital, and it seems to me to be quite wrong to count a patient twice because of some special arrangement requiring him or her to see two different officers.

If, however, a patient is treated in the casualty department and on some subsequent attendance for re-dressing is admitted as an in-patient, I think the case should be reckoned in both departments, as it received treatment, and therefore incurred expense, in the casualty department before becoming an in-patient, and this is necessary to differentiate the cost as between in- and out-patients.

### Another Provincial View.

By the Secretary of a Provincial General Hospital.

The answer to the question may possibly be found in the value placed by Boards of Management and Hospital Committees on the keeping of correct records. Where accuracy is expected Hospital Boards and Committees will expect to find records of initial treatment in the casualty registers. Too much stress cannot be laid upon this in view of the fact that Casualty Record Books now become important books of reference in connection with claims under the various Workmen's Compensation Acts. For general reasons therefore (it does not seem necessary to refer to such points as that the casualty department nursing staff, dressings, equipment, and appliances are used) it cannot be wrong to pass such patients through the casualty books. Possibly the only reason against such a course is the fact that the numbers of casualties are thus augmented. Such cases cannot be very numerous, and the objection is not a serious one.

### COMING EVENTS OF THE WEEK.

**Monday, April 25.**—Annual meeting, People's Refreshment House Association, Broadway Chambers, S.W., 2.30.

Meeting, Boys' Country Work Scheme, Grosvenor House, S.W., 3 p.m.

League of Mercy, Hitchin District, Old Town Hall, Hitchin, Miss Lucas and Miss Seebohm.

**Tuesday, April 26.**—St. Peter's Hospital Festival Dinner, Ritz Hotel, 7.30.

Zenana Medical Mission, Annual meeting, Portugal Street, Kingsway, 3 p.m.

League of Mercy, Chelsea District, Dramatic Entertainment organised by Mistress Vernon Harcourt, Royal Albert Hall Theatre, 8.30 p.m.

**Wednesday, April 27.**—Annual meeting, Invalid Children Aid Association, Sunderland House, Curzon Street, 3 p.m.

Annual meeting, London and Counties Medical Protection Society.

Annual meeting, Central London Throat and Ear Hospital, Gray's Inn Road, 4 p.m.

**Thursday, April 28.**—League of Mercy, North Kensington District, Mrs. G. K. Mills "At Home," 17 Stanley Crescent, W., 8.30 p.m.

**Friday, April 29.**—League of Mercy, North Kensington District, Lady Biddulph of Ledbury "At Home," 19 Ennismore Gardens, 3.30 p.m.

**Saturday, April 30.**—Annual dinner, French Hospital, Hotel Cecil, 7.30.

### The Institution Library.

"Hospital Expenditure: The Commissariat." For the use of Managers. ... ... ... ... ... 2s. 6d.
"The District Nursing Association; Hints on how to Start it." (Net, post free.) ... ... ... 1s. 1d.
"A Legal Handbook for the use of Hospital Authorities." .. ... ... ... ... ... ... 2s. 6d.
"Burdett's Hospitals and Charities," 1910. (Net, post free) ... ... ... ... ... ...10s. 11d.
Ledgers, Charts, Case-papers, and every kind of Institutional Literature.

Of all booksellers or of The Scientific Press, Limited, 28 and 29 Southampton Street, Strand, London, W.C.

